# Supplementary material for: In Silico Discovery and Evaluation of Inhibitors of the SARS-CoV-2 Spike Protein–HSPA8 Complex Towards Developing COVID-19 Therapeutic Drugs
Source: Viruses. 2024 Oct 31;16(11):1726. doi: 10.3390/v16111726 (PMC11599135; doi:10.3390/v16111726)
Supplement: Supplementary file 1 [file viruses-16-01726-s001.zip › viruses-3192034-supplementary.pdf]

viruses

MDPI

Supplementary Materials for

# *In Silico* Discovery and Evaluation of Inhibitors of the SARS–CoV–2 Spike Protein–HSPA8 Complex Towards Developing COVID-19 Therapeutic Drugs

**Liberty T. Navhaya [1], Thabe M. Matsebatlela [1], Mokgerwa Z. Monama [1] and Xolani H. Makhoba [2,*]**

[1] Department of Biochemistry, Microbiology, and Biotechnology, University of Limpopo, Turfloop campus, Sovenga 7270, South Africa; 202417284@keyaka.ul.ac.za (L.T.N.); thabe.matsebatlela@ul.ac.za (T.M.M.); mokgerwa.monama@ul.ac.za (M.Z.M.)
[2] Department of Life and Consumer Sciences, College of Agriculture and Environmental Sciences, University of South Africa (UNISA), Florida Campus, Roodepoort 1709, South Africa
* Correspondence: makhoxh@unisa.ac.za

**Note:** The material contained herein is supplementary to the article named in the title and published in the

International Journal of Molecular Sciences

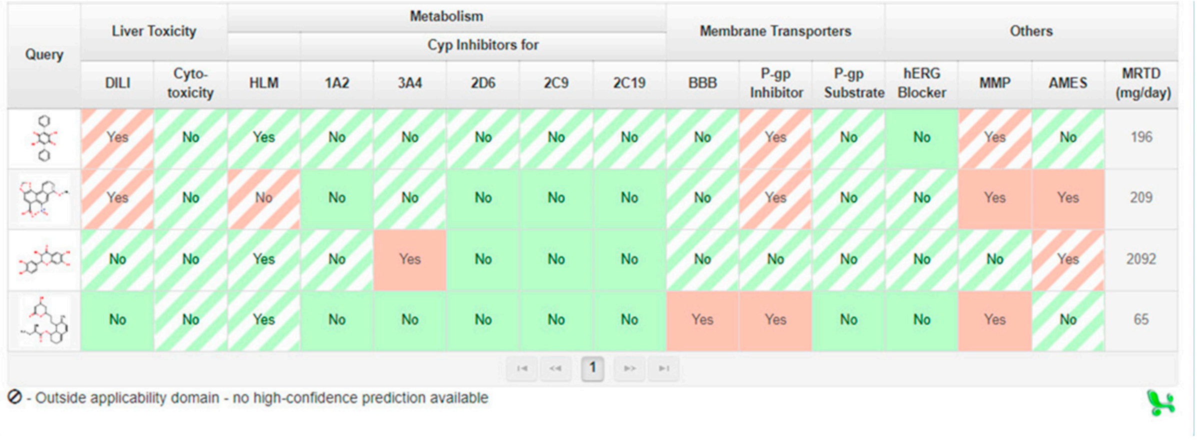

| Query | Liver Toxicity | | Metabolism | | | | | | | Membrane Transporters | | | Others | | | |
|---|---|---|---|---|---|---|---|---|---|---|---|---|---|---|---|---|
| | | | | Cyp Inhibitors for | | | | | | | | | | | | |
| | DILI | Cyto-toxicity | HLM | 1A2 | 3A4 | 2D6 | 2C9 | 2C19 | BBB | P-gp Inhibitor | P-gp Substrate | hERG Blocker | MMP | AMES | MRTD (mg/day) |
| | Yes | No | Yes | No | No | No | No | No | No | Yes | No | No | Yes | No | 196 |
| | Yes | No | No | No | No | No | No | No | No | Yes | No | No | Yes | Yes | 209 |
| | No | No | Yes | No | Yes | No | No | No | No | No | No | No | No | Yes | 2092 |
| | No | No | Yes | No | No | No | No | No | Yes | Yes | No | No | Yes | No | 65 |

⊘ - Outside applicability domain - no high-confidence prediction available

**Figure S1.** Results from the ADMET analysis of the four small molecules NSC44175, NSC11926, NSC36398, and NSC281245 [40]. The meaning of the colour coding is as follows: Green: desirable or optimal properties; Red: undesirable or poor properties; Red stripes: may pose risks or fail to meet desirable criteria; Green stripes: favourable properties.

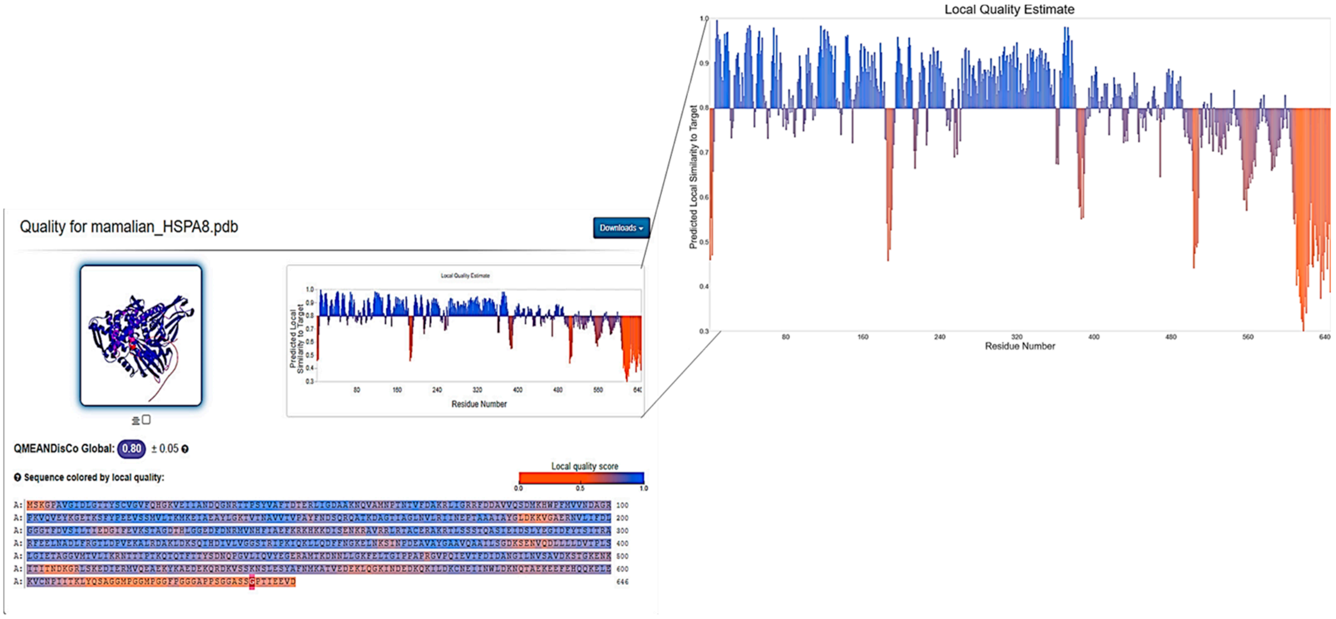


**Figure S2.** Qmean validation results of the modelled human HSPA8 [27].

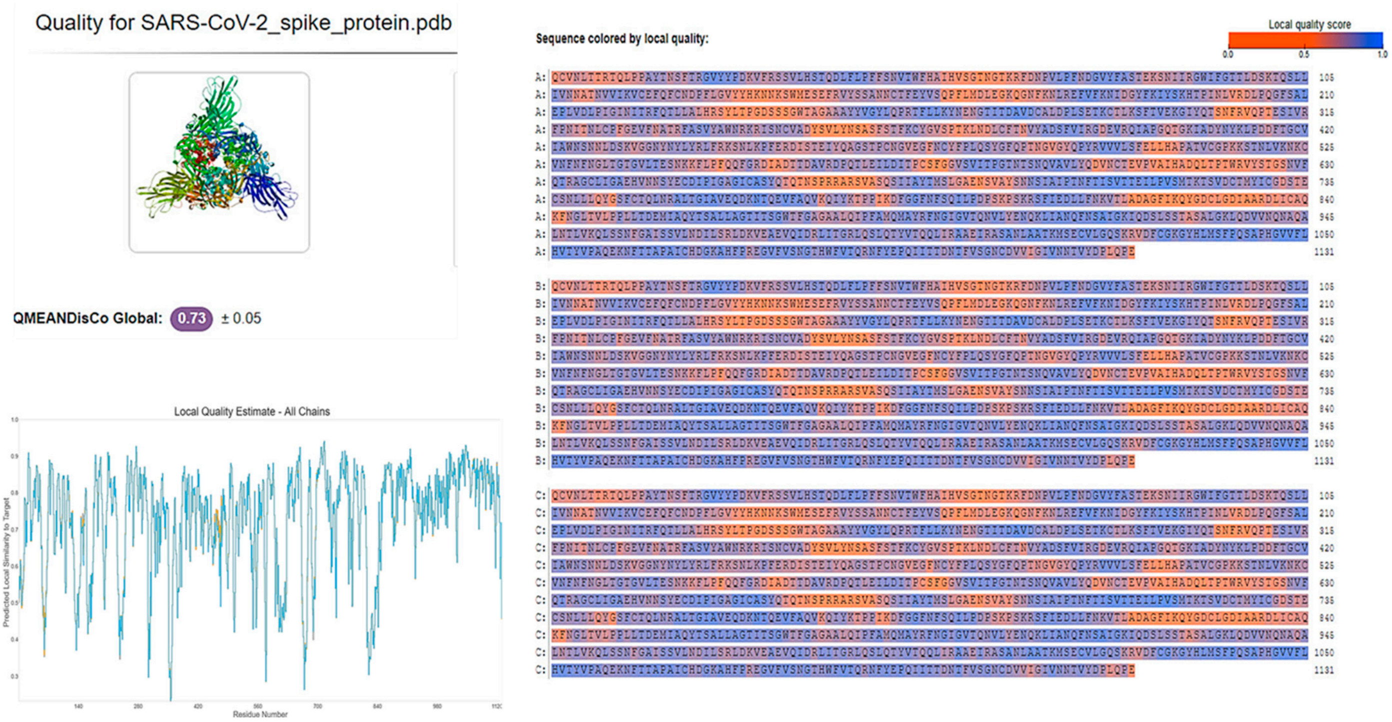


**Figure S3:** Qmean validation results of the modelled SARS-CoV-2 spike protein [27].

**Table S1:** Protein-protein docking best pose from Bioluminate4.6 (Maestro, Schrödinger suite) [31].

| Title | PIPER Cluster Size | PIPER pose energy (kcal/mol) | PIPER pose score | PIPER Model Number |
|---|---|---|---|---|
| prot-prot-docking_2_pose_1 | 65 | -748.301 | -101.189 | 11 |
| prot-prot-docking_2_pose_2 | 64 | -736.403 | -125.859 | 17 |
| prot-prot-docking_2_pose_3 | 50 | -622.428 | -156.643 | 736 |
| prot-prot-docking_2_pose_4 | 24 | -616.269 | -32.505 | 847 |
| prot-prot-docking_2_pose_5 | 22 | -694.401 | -39.79 | 86 |
| prot-prot-docking_2_pose_6 | 22 | -618.513 | -112.32 | 809 |
| prot-prot-docking_2_pose_7 | 19 | -635.099 | -33.974 | 519 |
| prot-prot-docking_2_pose_8 | 16 | -644.73 | -98.929 | 392 |
| prot-prot-docking_2_pose_9 | 16 | -867.403 | 337.213 | 2 |
| prot-prot-docking_2_pose_10 | 15 | -643.026 | -63.452 | 414 |
| prot-prot-docking_2_pose_11 | 15 | -820.341 | 105.533 | 3 |
| prot-prot-docking_2_pose_12 | 15 | -660.966 | 300.027 | 233 |
| prot-prot-docking_2_pose_13 | 13 | -617.408 | -141.853 | 826 |
| prot-prot-docking_2_pose_14 | 12 | -878.788 | 283.618 | 1 |
| prot-prot-docking_2_pose_15 | 12 | -796.446 | 73.679 | 5 |
| prot-prot-docking_2_pose_16 | 12 | -652.552 | -117.022 | 313 |
| prot-prot-docking_2_pose_17 | 12 | -624.942 | -91.458 | 682 |
| prot-prot-docking_2_pose_18 | 11 | -907.046 | 288.66 | 0 |
| prot-prot-docking_2_pose_19 | 11 | -699.018 | 310.646 | 75 |
| prot-prot-docking_2_pose_20 | 11 | -697.958 | 287.631 | 76 |
| prot-prot-docking_2_pose_21 | 11 | -641.266 | 387.979 | 434 |
| prot-prot-docking_2_pose_22 | 10 | -716.187 | 440.715 | 47 |
| prot-prot-docking_2_pose_23 | 10 | -702.358 | 179.046 | 67 |
| prot-prot-docking_2_pose_24 | 10 | -680.55 | 248.557 | 127 |
| prot-prot-docking_2_pose_25 | 10 | -659.919 | -109.081 | 246 |
| prot-prot-docking_2_pose_26 | 10 | -654.341 | 294.355 | 297 |
| prot-prot-docking_2_pose_27 | 10 | -637.711 | 98.584 | 489 |
| prot-prot-docking_2_pose_28 | 9 | -687.039 | -175.113 | 103 |
| prot-prot-docking_2_pose_29 | 7 | -661.378 | -49.428 | 228 |
| prot-prot-docking_2_pose_30 | 5 | -649.847 | 62.952 | 341 |

**Table S2:** Overall protein-protein interactions from the SARS-CoV-2 spike protein-HSPA8 complex [31].

| HSPA8 residues | Spike protein residues | Distance(A) | Specific Interactions | #HB |
|---|---|---|---|---|
| H:THR 411 | A:THR 478 | 1.6 | 1x hb to A:Thr 478 | 1 |
| H:PRO 468 | B:ASN 370 | 1.6 | 1x clash to B:Asn 370 | 0 |
| H:VAL 471 | B:SER 371 | 1.8 | | 0 |
| H:LYS 493 | B:LEU 335 | 1.8 | | 0 |
| H:THR 405 | A:THR 478 | 1.9 | | 0 |
| H:TYR 431 | A:PHE 486 | 1.9 | | 0 |
| H:GLN 426 | A:SER 477 | 1.9 | 1x hb to A:Ser 477 | 1 |
| H:TYR 431 | A:TYR 489 | 1.9 | | 0 |
| H:ALA 406 | A:GLY 485 | 1.9 | | 0 |
| H:ARG 469 | A:GLU 484 | 2.0 | 1x hb, 1x salt bridge to A: Glu 484 | 1 |
| H:PHE 428 | A:ASN 487 | 2.0 | 1x clash to A:Asn 487 | 0 |
| H:THR 429 | A:PHE 486 | 2.1 | 1x clash to A:Phe 486 | 0 |

| | | | | |
|---|---|---|---|---|
| H:ARG 469 | A:GLN 493 | 2.1 | 1x hb to A:Gln 493 | 1 |
| H:GLY 407 | A:VAL 483 | 2.2 | | 0 |
| H:THR 429 | A:TYR 489 | 2.2 | | 0 |
| H:ALA 406 | A:CYS 488 | 2.2 | | 0 |
| H:THR 429 | A:ALA 475 | 2.2 | | 0 |
| H:GLN 473 | B:ASP 364 | 2.2 | 1x hb to B:Asp 364 | 1 |
| H:ARG 469 | A:PHE 490 | 2.2 | | 0 |
| H:GLY 470 | A:PHE 456 | 2.2 | | 0 |
| H:ALA 406 | A:PHE 486 | 2.2 | | 0 |
| H:ALA 467 | B:SER 371 | 2.2 | | 0 |
| H:GLN 473 | B:VAL 367 | 2.2 | | 0 |
| H:VAL 438 | A:PHE 486 | 2.3 | | 0 |
| H:THR 405 | A:PHE 486 | 2.3 | | 0 |
| H:GLN 473 | B:LEU 335 | 2.3 | | 0 |
| H:TYR 431 | A:GLY 485 | 2.3 | | 0 |
| H:GLY 470 | A:TYR 489 | 2.3 | | 0 |
| H:ILE 403 | A:PHE 486 | 2.3 | | 0 |
| H:PRO 468 | B:SER 371 | 2.3 | | 0 |
| H:GLU 404 | A:PHE 486 | 2.4 | | 0 |
| H:THR 430 | A:TYR 489 | 2.4 | | 0 |
| H:VAL 471 | B:VAL 367 | 2.4 | | 0 |
| H:VAL 409 | A:ASN 481 | 2.4 | | 0 |
| H:ASP 433 | A:GLN 493 | 2.4 | 1x hb to A:Gln 493 | 1 |
| H:LYS 493 | B:THR 333 | 2.5 | 1x hb to B:Thr 333 | 1 |
| H:VAL 409 | A:PRO 479 | 2.5 | | 0 |
| H:THR 411 | A:SER 477 | 2.5 | | 0 |
| H:THR 429 | A:ASN 487 | 2.5 | | 0 |
| H:ARG 469 | A:PHE 456 | 2.5 | | 0 |
| H:THR 405 | A:ASN 487 | 2.6 | | 0 |
| H:VAL 471 | B:ASN 370 | 2.6 | | 0 |
| H:VAL 409 | A:THR 478 | 2.6 | | 0 |
| H:ILE 440 | A:PHE 486 | 2.6 | | 0 |
| H:GLU 475 | B:THR 333 | 2.7 | | 0 |
| H:SER 494 | B:GLY 339 | 2.7 | | 0 |
| H:LYS 493 | B:ASN 334 | 2.8 | | 0 |
| H:MET 410 | A:THR 478 | 2.8 | | 0 |
| H:PHE 428 | A:THR 478 | 2.8 | | 0 |
| H:ALA 406 | A:VAL 483 | 2.9 | | 0 |
| H:ALA 406 | A:ASN 487 | 2.9 | | 0 |
| H:SER 494 | B:PRO 337 | 2.9 | | 0 |
| H:THR 427 | A:GLY 476 | 2.9 | | 0 |
| H:PHE 428 | A:PHE 486 | 3.0 | | 0 |
| H:ALA 406 | A:GLU 484 | 3.0 | | 0 |
| H:THR 411 | A:PRO 479 | 3.0 | | 0 |
| H:ARG 469 | A:LEU 492 | 3.0 | | 0 |
| H:THR 430 | A:PHE 486 | 3.1 | | 0 |
| H:THR 425 | B:THR 333 | 3.1 | | 0 |
| H:PRO 465 | B:ASN 343 | 3.1 | | 0 |
| H:ARG 469 | A:LEU 455 | 3.2 | | 0 |
| H:ARG 469 | A:TYR 489 | 3.2 | | 0 |
| H:SER 494 | B:CYS 336 | 3.2 | | 0 |

| | | | |
|---|---|---|---|
| H:MET 410 | A:PRO 479 | 3.2 | 0 |
| H:VAL 409 | A:CYS 480 | 3.3 | 0 |
| H:GLY 407 | A:GLY 485 | 3.3 | 0 |
| H:SER 494 | B:PHE 338 | 3.3 | 0 |
| H:PRO 466 | B:ASN 343 | 3.4 | 0 |
| H:SER 494 | B:GLU 340 | 3.4 | 0 |
| H:VAL 476 | A:PHE 486 | 3.4 | 0 |
| H:GLN 426 | A:GLY 476 | 3.4 | 0 |
| H:PRO 468 | A:GLN 493 | 3.5 | 0 |
| H:GLN 426 | A:THR 478 | 3.5 | 0 |
| H:LYS 493 | B:PRO 337 | 3.6 | 0 |
| H:PHE 428 | A:GLY 476 | 3.6 | 0 |
| H:PRO 468 | A:LEU 455 | 3.6 | 0 |
| H:VAL 471 | A:PHE 456 | 3.6 | 0 |
| H:THR 427 | A:ALA 475 | 3.6 | 0 |
| H:VAL 412 | A:SER 477 | 3.7 | 0 |
| H:THR 495 | B:GLU 340 | 3.7 | 0 |
| H:LYS 493 | B:CYS 336 | 3.7 | 0 |
| H:ALA 406 | A:CYS 480 | 3.7 | 0 |
| H:THR 427 | A:ASN 487 | 3.7 | 0 |
| H:ARG 469 | B:ASN 370 | 3.8 | 0 |
| H:ILE 474 | A:PHE 486 | 3.8 | 0 |
| H:PRO 465 | B:GLY 339 | 3.8 | 0 |
| H:THR 411 | A:GLY 476 | 3.8 | 0 |
| H:LYS 493 | B:VAL 362 | 3.9 | 0 |

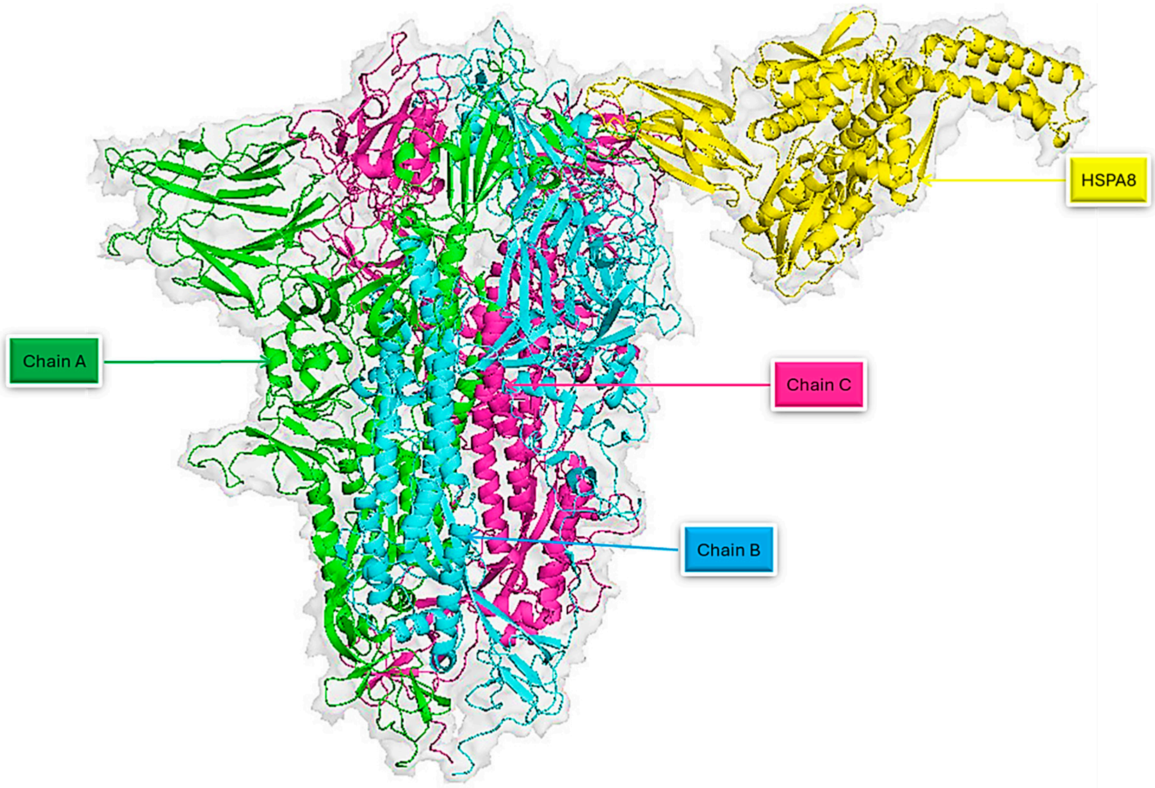


**Figure S4:** Structural insight into the HSPA8:spike protein complex: A BioLuminate-derived protein-protein docking study revealing intriguing interactions [31,44].

**Table S3:** Sitescore, dscore, volume, exposure, enclosure, contact, phobic, philic and balance of human HSPA8 and SARS-CoV-2 spike protein from the identified active sites obtained from SiteMap [33–35].

| Protein | Site | SiteScore | Size | Dscore | Volume | Exposure | Enclosure | Contact | Phobic | Philic | Balance |
|---|---|---|---|---|---|---|---|---|---|---|---|
| **HSPA8** | 1 | 1.114 | 230 | 0.935 | 341.2854 | 0.307 | 0.868 | 1.128 | 0.301 | 1.614 | 0.186 |
| | 2 | 1.031 | 165 | 0.922 | 407.827 | 0.531 | 0.744 | 0.971 | 0.181 | 1.423 | 0.127 |
| | 3 | 1.005 | 100 | 0.991 | 288.806 | 0.543 | 0.706 | 0.965 | 0.318 | 1.142 | 0.278 |
| | 4 | 0.927 | 87 | 0.940 | 166.698 | 0.571 | 0.655 | 0.859 | 0.542 | 1.012 | 0.536 |
| | 5 | 0.835 | 64 | 0.745 | 161.896 | 0.579 | 0.671 | 0.892 | 0.136 | 1.257 | 0.108 |
| | | | | | | | | | | | |
| **Spike protein** | 4 | 1.105 | 877 | 1.148 | 3367.574 | 0.507 | 0.791 | 0.936 | 0.990 | 0.817 | 1.212 |
| | 3 | 1.057 | 916 | 1.034 | 2404.773 | 0.448 | 0.783 | 0.992 | 0.434 | 1.151 | 0.377 |
| | 5 | 1.054 | 715 | 1.034 | 2190.398 | 0.516 | 0.779 | 1.017 | 0.570 | 1.148 | 0.497 |
| | 2 | 1.033 | 966 | 1.031 | 2715.188 | 0.535 | 0.748 | 0.929 | 0.419 | 1.097 | 0.382 |
| | 1 | 1.031 | 1344 | 1.052 | 4515.595 | 0.582 | 0.734 | 0.897 | 0.439 | 1.006 | 0.437 |
| | | | | | | | | | | | |
| **HSPA8:spike complex** | 4 | 1.096 | 875 | 1.135 | 3361.057 | 0.516 | 0.786 | 0.925 | 0.902 | 0.849 | 1.063 |
| | 5 | 1.049 | 682 | 1.03 | 2181.48 | 0.553 | 0.771 | 0.993 | 0.648 | 1.144 | 0.566 |
| | 1 | 1.042 | 1174 | 1.044 | 3309.95 | 0.508 | 0.761 | 0.933 | 0.541 | 1.082 | 0.5 |
| | 2 | 1.037 | 1126 | 1.03 | 3186.813 | 0.514 | 0.753 | 0.93 | 0.438 | 1.109 | 0.395 |
| | 3 | 1.035 | 1086 | 1.036 | 3155.6 | 0.521 | 0.751 | 0.928 | 0.443 | 1.086 | 0.408 |

**Table S4:** Amino acid residues located on the most preferred binding sites of human HSPA8, SARS-CoV-2 spike protein, and HSPA8:spike protein complex [34,45].

| Amino acid residues on the most preferred binding site of human HSPA8. | Amino acid residues on the most preferred binding site of the SARS-CoV-2 spike protein. | Amino acid residues on the most preferred binding site of the HSPA8:spike protein complex |
|---|---|---|
| ASP10, LEU11, GLY12, THR13, THR14, TYR15, SER16, CYS17, THR37, PRO39, SER40, TYR41, LYS56, LYS71, THR145, PRO147, ALA148, GLU175, PRO176, ASH199, LEU200, GLY201, GLY202, GLY203, THR204, PHE205, ASP206, GLY230, GLU231, ASP234, ARG261, ARG264, THR265, GLU268, LYS271, ARG272, SER275, VAL337, GLY338, GLY339, SER340, ARG342, ILE343, PRO365, ASP366, GLU367, ALA368, VAL369, ALA370, TYR371 | GLU725, ILE726, LEU727, PRO728, MET731, THR732, GLN762, LEU763, ARG765, ALA766, LEU767, GLY769, ILE770, VAL772, GLU773, GLN774, LYS776, GLU780, VAL781, GLN784, GLY946, LYS947, LEU948, ASP950, VAL951, GLN954, ASN955, GLN957, ALA958, LEU959, THR961, LEU962, GLN965, SER1003, THR1006, TYR1007, GLN1010, GLN1011, LEU1012, ILE1013, ARG1014, ALA1015, ALA1016, GLU1017, ILE1018, ARG1019, ALA1020, SER1021, ALA1022, ASN1023, LEU1024, ALA1026, THR1027, LYS1028, SER1030, GLU1031, LEU1034, ARG1039, VAL1040, ASP1041, | THR723, GLU725, ILE726, LEU727, PRO728, MET731, THR732, PHE759, GLN762, LEU763, ARG765, ALA766, LEU767, GLY769, ILE770, VAL772, GLU773, GLN774, LYS776, GLU780, GLN784, SER946, LYS947, LEU948, ASP950, VAL951, LEU952, LEU953, GLN954, ASN955, GLN957, ALA958, THR961, LEU962, GLN965, SER1003, GLN1005, THR1006, TYR1007, VAL1008, GLN1010, GLN1011, LEU1012, ILE1013, ARG1014, ALA1015, ALA1016, GLU1017, ILE1018, ARG1019, ALA1020, SER1021, ALA1022, ASN1023, LEU1024, ALA1026, THR1027, LYS1028, SER1030, GLU1031, LEU1034, ARG1039, VAL1040, ASP1041, PHE1042, CYX1043, GLY1044, LYS1045, HIE1064 |

| |
|---|
| PHE1042, CYX1043, GLY1044, LYS1045, HIE1064 |

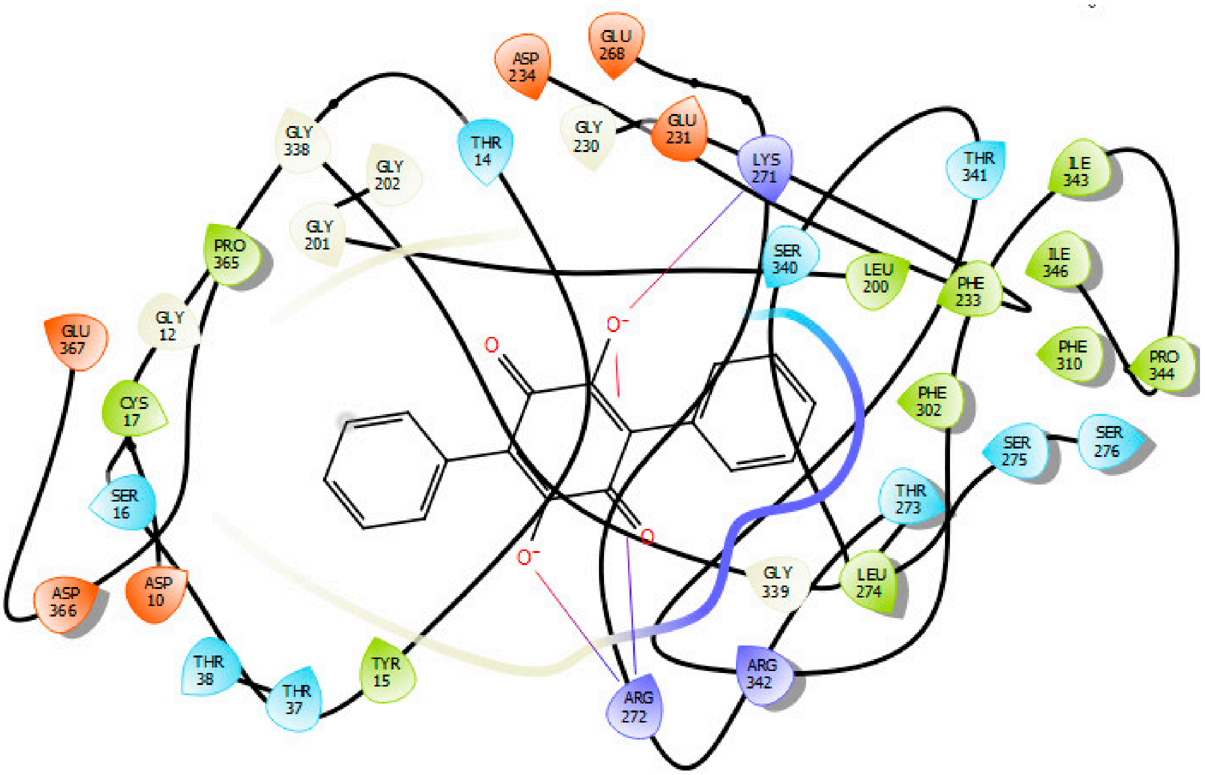


**Figure S5:** Ligand interaction diagram of the HSPA8 showing the extra salt bridge interactions with the ligand NSC44175 [47–49].

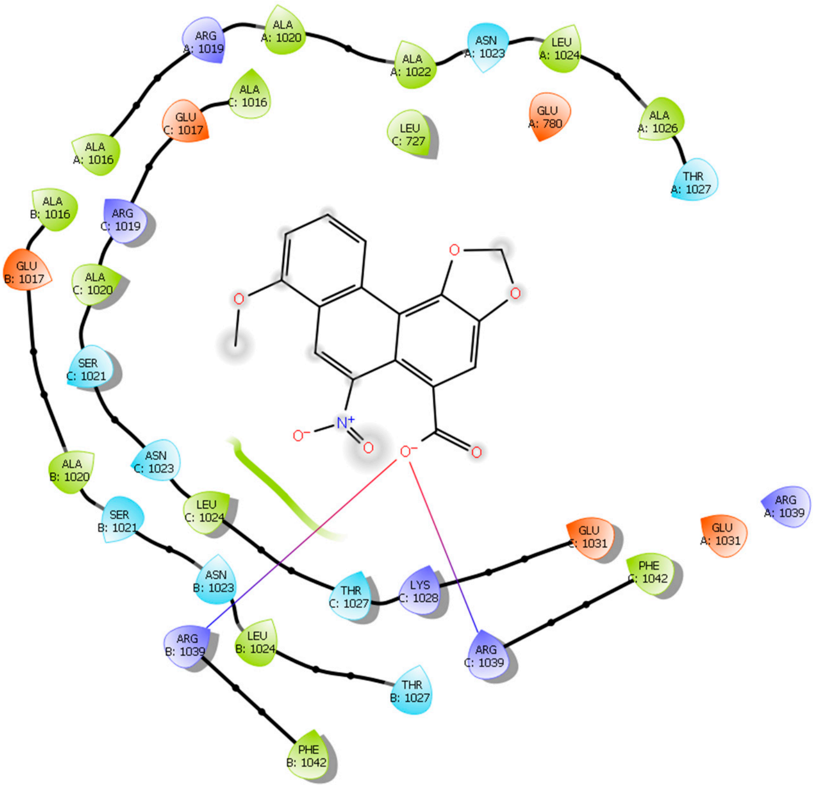

**Figure S6:** Ligand interaction diagram of the protein complex, HSPA8:spike protein, showing the additional salt bridge interactions with NSC11926 [38,48,49].
